# Receptor for advanced glycation end-products and World Trade Center particulate induced lung function loss: A case-cohort study and murine model of acute particulate exposure

**DOI:** 10.1371/journal.pone.0184331

**Published:** 2017-09-19

**Authors:** Erin J. Caraher, Sophia Kwon, Syed H. Haider, George Crowley, Audrey Lee, Minah Ebrahim, Liqun Zhang, Lung-Chi Chen, Terry Gordon, Mengling Liu, David J. Prezant, Ann Marie Schmidt, Anna Nolan

**Affiliations:** 1 Department of Medicine, Division of Pulmonary, Critical Care and Sleep Medicine, New York University School of Medicine, New York, New York, United States of America; 2 Department of Respiratory Medicine, PLA, Army General Hospital, Beijing, China; 3 Department of Environmental Medicine, New York University School of Medicine, New York, New York, United States of America; 4 Department of Population Health, Division of Biostatistics, New York University School of Medicine, New York, New York, United States of America; 5 Bureau of Health Services and Office of Medical Affairs, Fire Department of New York, Brooklyn, New York, United States of America; 6 Department of Medicine, Pulmonary Medicine Division, Montefiore Medical Center and Albert Einstein College of Medicine, Bronx, New York, United States of America; 7 Departments of Biochemistry and Molecular Pharmacology and Pathology, Division of Endocrinology, New York University School of Medicine, New York, New York, United States of America; Forschungszentrum Borstel Leibniz-Zentrum fur Medizin und Biowissenschaften, GERMANY

## Abstract

World Trade Center-particulate matter(WTC-PM) exposure and metabolic-risk are associated with WTC-Lung Injury(WTC-LI). The receptor for advanced glycation end-products (RAGE) is most highly expressed in the lung, mediates metabolic risk, and single-nucleotide polymorphisms at the AGER-locus predict forced expiratory volume(FEV). Our objectives were to test the hypotheses that RAGE is a biomarker of WTC-LI in the FDNY-cohort and that loss of RAGE in a murine model would protect against acute PM-induced lung disease. We know from previous work that early intense exposure at the time of the WTC collapse was most predictive of WTC-LI therefore we utilized a murine model of intense acute PM-exposure to determine if loss of RAGE is protective and to identify signaling/cytokine intermediates. This study builds on a continuing effort to identify serum biomarkers that predict the development of WTC-LI. A case-cohort design was used to analyze a focused cohort of male never-smokers with normal pre-9/11 lung function. Odds of developing WTC-LI increased by 1.2, 1.8 and 1.0 in firefighters with soluble RAGE (sRAGE)≥97pg/mL, CRP≥2.4mg/L, and MMP-9≤397ng/mL, respectively, assessed in a multivariate logistic regression model (ROC_AUC_ of 0.72). Wild type(WT) and RAGE-deficient(Ager^*-/-*^*)* mice were exposed to PM or PBS-control by oropharyngeal aspiration. Lung function, airway hyperreactivity, bronchoalveolar lavage, histology, transcription factors and plasma/BAL cytokines were quantified. WT-PM mice had decreased FEV and compliance, and increased airway resistance and methacholine reactivity after 24-hours. Decreased IFN-γ and increased LPA were observed in WT-PM mice; similar findings have been reported for firefighters who eventually develop WTC-LI. In the murine model, lack of RAGE was protective from loss of lung function and airway hyperreactivity and was associated with modulation of MAP kinases. We conclude that in a multivariate adjusted model increased sRAGE is associated with WTC-LI. In our murine model, absence of RAGE mitigated acute deleterious effects of PM and may be a biologically plausible mediator of PM-related lung disease.

## Introduction

Obstructive airway disease (OAD) due to particulate matter (PM) exposure is a major health concern worldwide.[1–3] During the events of September 11, 2001, Fire Department of New York City (FDNY) firefighters were exposed to World Trade Center-particulate matter (WTC-PM), a known cause of lung function loss. [4–9] In WTC-PM exposed firefighters, metabolically active biomarkers have been associated with the development of OAD.[10–15] Increasing evidence supports the importance of the receptor for advanced glycation end-products (RAGE), also known as the advanced glycation end-product receptor (AGER), in OAD. However, mechanisms of PM-associated lung disease and the role of RAGE are not well characterized.

RAGE is a member of the immunoglobulin super family, exists in many isoforms and binds diverse ligands including products of metabolic stress such as AGEs, High Mobility Group Box-1(HMGB1), S100 and amyloid-β peptides. The membrane bound form, generally referred to as RAGE or AGER, has been shown to be a key mediator in many chronic conditions including inflammation, vascular injury and metabolic syndrome. [16–18] Soluble forms of RAGE can be formed by variations in splicing or cleavage by metalloproteinases, including ADAM10 and MMP-9; total soluble RAGE includes all soluble isoforms and is traditionally denoted by sRAGE and may act as a decoy receptor for RAGE ligands. [19–21] Furthermore, the utility of sRAGE as a diagnostic biomarker in emphysema and chronic inflammatory diseases is currently being explored. [22, 23]

In most end organs RAGE is expressed at low baseline levels and increases with disease. RAGE is expressed at the highest baseline level in the lung, and is found in alveolar type epithelial cells, vascular endothelial cells, alveolar macrophages and the smooth muscle cells of the airways.[21, 24] It specifically localizes in the adult lung on the basolateral membrane of alveolar type-1 epithelial cells.[25] Conflicting data exist on the directionality of RAGE and sRAGE expression in lung disease. Increased levels of sRAGE predicted poor fluid clearance in acute lung injury (ALI).[26] In a direct ALI model elevated sRAGE levels were seen in bronchoalveolar lavage (BAL) 24 hours after LPS injury, while treatment with mouse recombinant sRAGE 1 hour after injury attenuated neutrophilic infiltration, inflammatory mediators and lung permeability.[27] In indirect models of lung injury, such as murine transfusion related lung injury, there was no elevation of BAL levels of RAGE.[28] In subjects with OAD, explanted lung was found to have both increased expression and BAL levels of RAGE.[29, 30] Airway inflammation in OAD is associated with reduced levels of circulating sRAGE.[31, 32] Furthermore, RAGE has been implicated in a murine smoke exposure model of emphysema and a more recent review highlights the role of sRAGE as a biomarker of OAD.[22, 33, 34]

The role of RAGE has been examined in several occupational lung diseases. RAGE expression has been shown to be depleted in the fibrotic lung.[35, 36] In a bleomycin model of pulmonary fibrosis Ager^-/-^ are protected.[37] In contrast, in a murine model of silicosis, mice deficient of Ager had a differing pattern of fibrosis but there was no effect on the severity of fibrosis after a single intratracheal instillation of silica.[38] In both house dust mite and ovalbumin models of asthma, Ager^-/-^ were protected from airway hyperreactivity. Furthermore, these findings were recapitulated with Ager inhibition.[39]

Single nucleotide polymorphisms within the AGER locus have been associated with FEV_1_ in two genome-wide association studies.[40, 41] More recently several groups have correlated AGER associated loci in *in vitro* models to further our understanding of possible mechanisms. The promoter variant AGER-429 T/C (rs1800625) was associated with the severity of cystic fibrosis.[42–44] In addition, they found that cells with the functional promoter AGER-429C had increased RAGE expression.[42] In a population of smokers the rs2070600T (Ser82) allele was associated with higher FEV_1_ and FEV_1_/FVC and lower sRAGE levels. Overexpression of Ser82 in an airway epithelium model resulted in lower sRAGE elaboration.[45]

Finally, our group has identified elevated serum lysophosphatidic acid (LPA), a product of low-density lipoprotein (LDL) and known ligand of RAGE, as a WTC-LI biomarker in the FDNY-cohort.[11, 46, 47] Here we investigate if sRAGE is a WTC-LI biomarker in the FDNY WTC exposed-cohort. Our prior *in vitro* work showed that WTC-PM exposure mediated an inflammatory phenotype 24 hours after exposure.[48] Since intense early exposure to WTC-PM is a significant predictor of later loss of lung function we have chosen to use a single high dose exposure in our murine PM exposure model.[6] Prior work showed that WTC-PM administered by oropharyngeal aspiration recruits neutrophils to the lung within 24 hours and causes airway hyperresponsiveness, findings also seen in the human WTC-exposed cohort at later time points.[5, 6, 49, 50] The current investigation utilizes a murine PM aspiration model to determine if lack of RAGE (Ager) is protective against acute lung function loss and airway hyperreactivity following WTC-PM exposure.

## Methods

### Ethics statement

Before enrollment subjects signed informed consents that were approved by the institutional review board (IRB) of Montefiore Medical Center (#07-09-320) for serum banking and the current study was further approved by the New York University (NYU) IRB (#11–00439). Murine experiments were reviewed and approved by the NYU IACUC # s16-00447.

### WTC FDNY biomarker cohort

As previously described, all subjects exposed to WTC-PM were enrolled in the Medical Monitoring Treatment Program (MMTP) within 6 months of 9/11/2001 (9/11). At their MMTP visit spirometry was performed and serum was collected and processed as previously described.[10, 11, 51–53] A subset of n = 1720 presented with pulmonary symptoms between 9/11/2001 and March 2008 and were referred to subspecialty pulmonary evaluation (SPE) which included pulmonary function testing.

**Fig 1:**
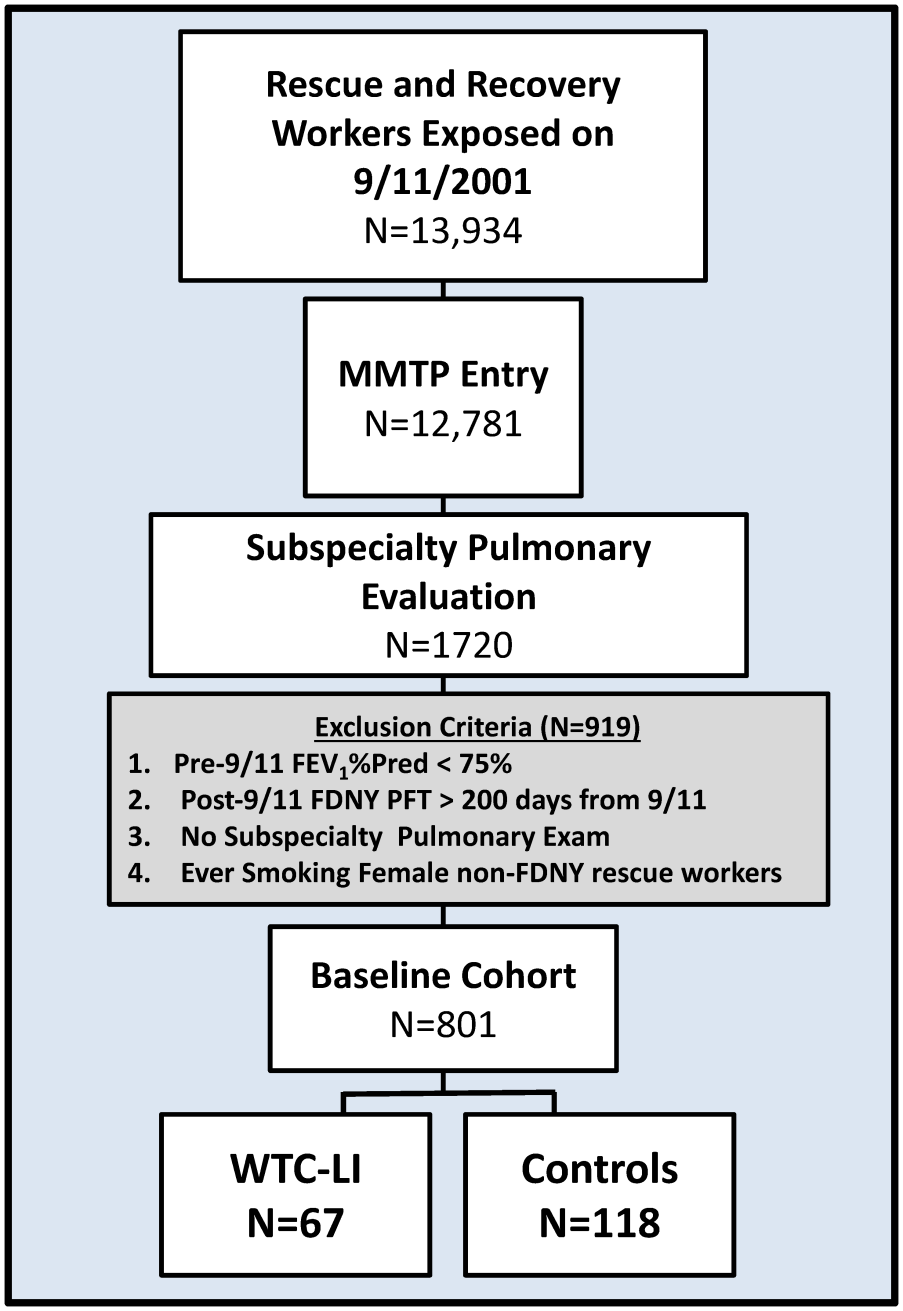
Case-cohort design. Of the 13,934-exposed rescue and recovery workers, 92% enrolled in the MMTP. A subset of n = 1720 experienced pulmonary symptoms and had a subspecialty pulmonary evaluation (SPE) before March 2008. Exclusion criteria were applied to form a baseline cohort of N = 801. Those with all biomarkers available were (n = 67) WTC-LI cases; and (n = 118) controls.

Similar to prior work, we used a case-cohort design to determine associations of early serum biomarkers obtained at MMTP with FEV_1_% predicted<lower limit of normal (LLN; <5^th^-percentile of predicted) at SPE, defined as WTC-LI.[10, 52, 54] The case-cohort design is a cost-effective sampling design within large cohorts, and the controls can serve as a universal control group for every outcome in the baseline cohort from which is was drawn, Fig 1.[55–58] Subjects were included in the baseline cohort for this study if they were never-smoking male firefighters who had reliable National Health and Nutrition Examination Survey (NHANES) normative data for predicted FEV_1_, post-9/11 FDNY PFTs within 200 days of 9/11, and pre-9/11 FEV1 >75% predicted (n  =  801 (47%) out of 1720).[59–66] The control (N  =  171) was randomly selected from the baseline cohort after stratification on BMI and FEV_1_ at MMTP entry. A complete data set including serum was available for n  =  118 controls and n  =  67 cases, Fig 1 and S1 Table.

### Murine oropharyngeal aspiration model

Female wild-type (WT) C57Bl/6 mice > 12 weeks old (Jackson Laboratory) were age- and weight-matched to mice that lacked RAGE (Ager^-/-^; Ager refers to the murine gene while AGER refers to the human gene and the protein in humans and mouse) on a C57Bl/6 background (Ann Marie Schmidt).[46, 49] Mice had free access to food/water and 12-hour light/dark cycles. WTC-PM was obtained, as previously described, from 5 locations within 0.5 miles of Ground Zero on 9/13/01.[49, 67] Composition was determined by x-ray fluorescence analysis using techniques as previously published, S2 Table.[67] Oropharyngeal aspiration, equivalent to intratracheal instillation in deposition efficiency, was used to deliver PM as previously described.[48, 49, 68] Mice aspirated 100μg-WTC-PM_53_ suspended in sterile-PBS or isovolemic sterile-PBS (Fisher). Mice that were littermates and cohoused were exposed to both PBS and PM on the same day to avoid batch bias. After 24 hours, mice were sequentially analyzed on *flexiVent*–mice were excluded from further analysis if cessation of normal tidal breathing before *flexiVent* was observed. Experiments were repeated until a minimum of 5 mice per exposure group was obtained. A single, 100μg dose of WTC-PM was chosen due to its estimated ability to cause similar adverse pulmonary effects in mice to those seen in a rescue-worker exposed to 425 μg/m^3^ of WTC-PM over an 8-hour shift.[49] This rescue worker exposure level falls within measured concentrations of PM at the 9/11 debris pile.[69]

### Murine lung mechanics

**Fig 2:**
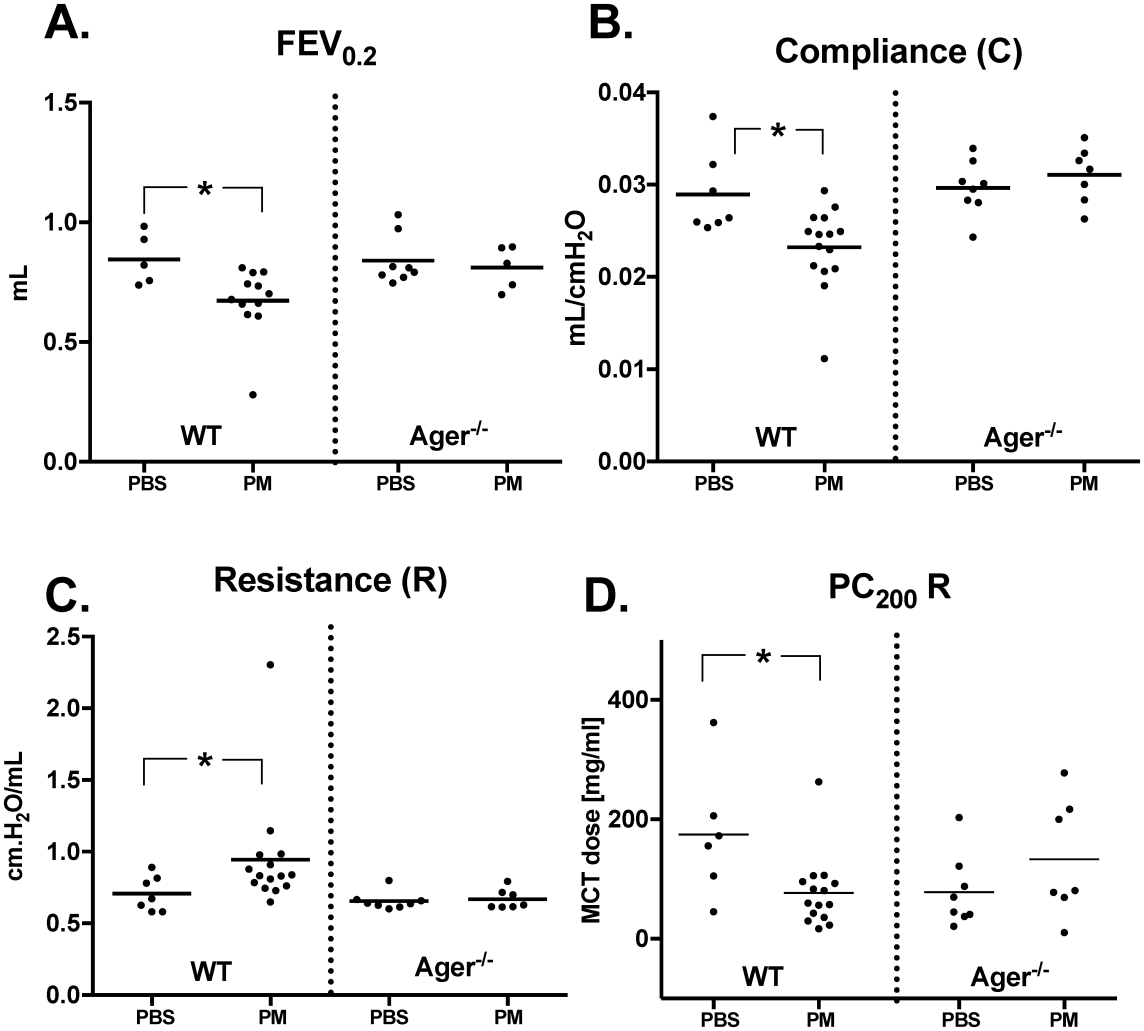
Age*r* ^-/-^ mice are protected from loss of lung function 24 hours after WTC-PM exposure. *24 Hours after* a single exposure to WTC-PM, WT mice show significant differences in (A) FEV_0.2_ (B) compliance and (C) resistance compared to PBS controls. These parameters did not differ between Ager^-/-^ mice exposed to PM and their PBS controls. (D) Airway Hyperreactivity (PC_200_): WT-PM mice exhibited hyperreactivity, whereas Ager^-/—^PM did not. A total of WT-PBS = 7, WT-PM = 15, Ager^-/—^PBS = 8, and Ager ^-/—^PM = 7 mice were analyzed. WT-PBS = 2, WT-PM = 3 and Ager^-/—^PM = 2 were excluded from FEV_0.2_ analyses as they did not meet standards outlined in the methods. Additionally, WT-PBS = 1 was excluded from methacholine analysis due to a dosing error.

**Fig 3:**
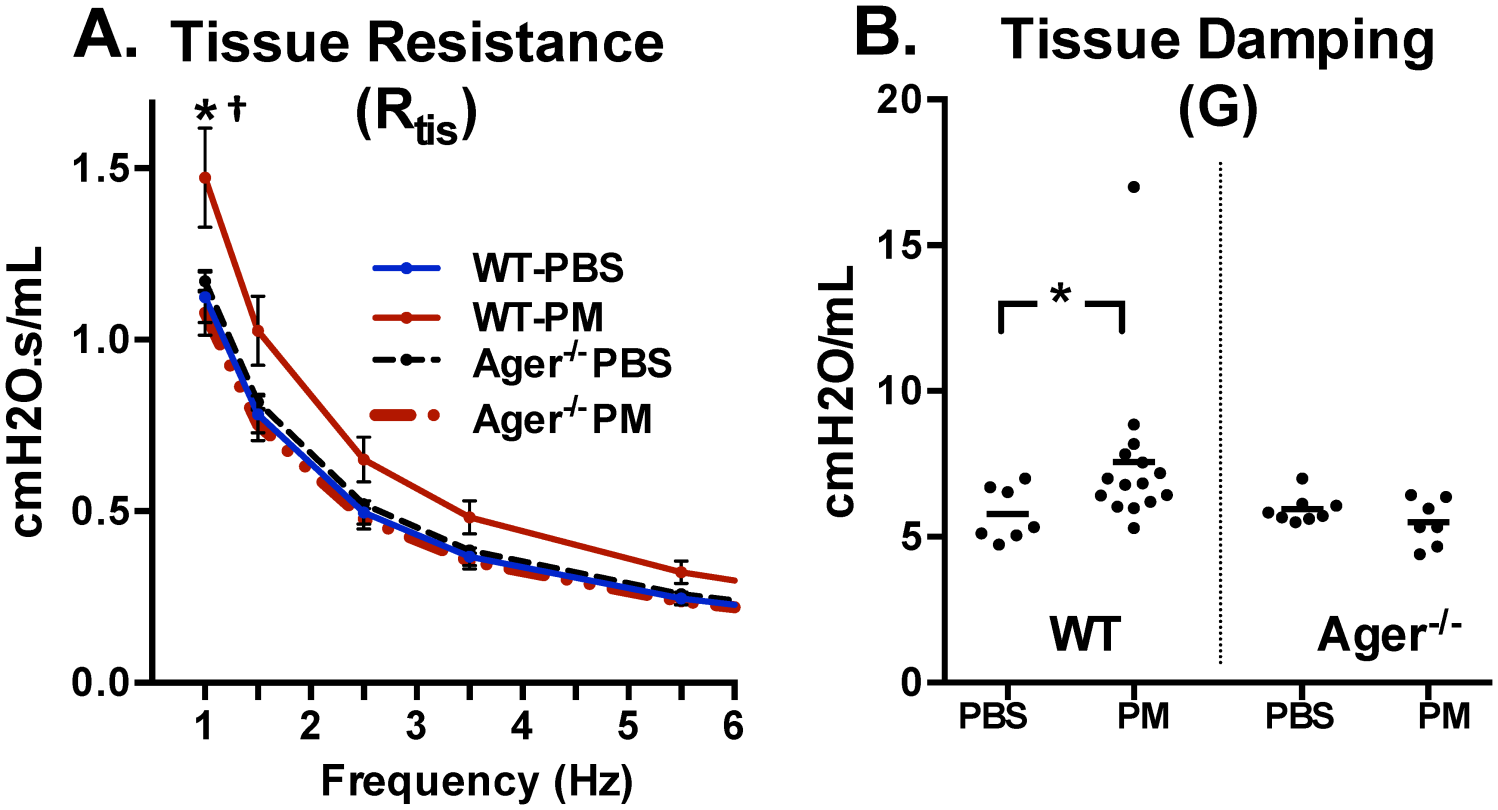
PM exposure affects small airways to a greater degree in WT compared to Ager^-/-^ mice. (A) WT-PM mice had significantly elevated tissue resistance at 1Hz p<0.001 (*). Ager^-/—^PM had significantly decreased tissue resistance at 1Hz (†) compared the Ager^-/—^PBS. (B) WT-PM, but not Ager^-/—^PM mice had significantly (*) higher tissue damping compared to controls. N ≥5 mice per group. WT-PBS = 7, WT-PM = 15, Ager^-/—^PBS = 8, and Ager^-/—^PM = 7 mice were analyzed.

*flexiVent*-FX1 (SCIREQ) was used to measure lung function.[70–79] Mice were anesthetized by intraperitoneal injection (0.11ml/10g) with Ketamine/Xylazine (100/10mg/ml, Troy-Laboratories) and tracheostomized with an 18G stainless-steel cannula (BD). Mice were connected to the *flexiVent* system by 18 G endotracheal cannula and placed in a whole-body plethysmograph, as previously described. [70–72] Mice were ventilated at a tidal volume of 10mL/kg, frequency of 150 breaths per minute with a PEEP of 3 cmH_2_0. Baseline lung mechanics measures were made using perturbations incorporated into automated scripts to ensure reproducibility. Each perturbation has its own internal quality control in which the obtained impedance data’s fit to the respective model is assessed and a coefficient of determination (COD) value is determined. N’s were included in the Figure Legends to reflect exclusions made based on these internal quality controls. The automated script used for baseline lung mechanics data collection in this experiment included three rounds of the following perturbations: Deep Inflation, Snapshot-150, Quick Prime-3, Pressure-Volume curve, and Negative Pressure Driven Forced Expiration. Averages of the three raw data points obtained were calculated for each subject and used in all further analyses seen in Figs 2 and 3B and S3 Table.

In the deep inflation perturbation lungs were inflated to 27cmH_2_O and held for 3 seconds; characteristic pressure/volume (PV) tracings were identified to confirm proper cannula placement and the absence of leaks.

Snapshot-150 and Quick Prime-3 are Forced Oscillation Technique (FOT) perturbations. The forced oscillation technique involves the delivery of pre-defined oscillatory airflow waveforms at the subject’s airway opening, specifically the opening of the endotracheal cannula, and analyzes pressure and volume signals to estimate parameters of lung function. Snapshot-150 delivered a single frequency forced oscillation (2.5 Hz, 1.2seconds) and pressure and volume signals were fit to a single compartment model to approximate resistance (R), elastance (E) and compliance (C) of the whole respiratory system (airways, lungs and chest wall), Fig 2 and S3 Table. All data included had a COD ≥0.98. Quick Prime-3 delivered broadband frequency forced oscillation (1–20.5 Hz, 3seconds) and pressure and volume signals were fit to a constant phase model to approximate Newtonian Resistance, also referred to as inertia of air (R_n_), Tissue Damping (G), and Tissue elastance (H), Fig 3 and S3 Table. All data included had a COD ≥0.91. Tissue resistance (R_tis_), was determined by iteratively fitting the real-portion of the input impedance data to the constant phase model, Fig 3A.[76, 80]

A step-wise pressure driven perturbation is used to generate a PV curve from which Quasi-static compliance (C_st_), hysteresis (Area), and Salazar-Knowles parameters A (maximal vital (total) lung capacity) and K (form of the deflecting PV Loop) were obtained, S3 Table.

Negative Pressure Forced Expiration (NPFE), a *flexiVent* hardware add-on, allows the study of expiratory flow limitations. A negative pressure reservoir is attached to a computer-controlled valve to generate a forced expiratory flow from the subject. During the execution of the NPFE manoeuver, the subject was inflated to a total lung capacity (TLC) state (30cmH_2_O over 1 second) and held at this pressure for 2 seconds after which a shutter valve connecting the mouse’s lungs to a negative pressure reservoir (held at -55 cmH_2_O) was opened. Flow-volume loops, peak expiratory flow (PEF), forced vital capacity (FVC), forced expired volumes (FEVx) and flows (FEFx) at user defined times are calculated by the software, Fig 2A and S3 Table. NPFE data was excluded when reservoir pressure variation exceeded 10%. [70–72]

### Murine hyperreactivity

Methacholine was diluted in sterile saline to 0, 6.25, 12.5, 25, 50, 100, 200mg/ml (Santa Cruz). Each dose was delivered over 10 seconds by a nebulizer extension (Aerogen ANP-1100) and Mdel, the dose delivered to the subject in μg, was calculated by the software.[68, 75, 81] An automated script was used after each dose of methacholine in which 11 SnapShot-150 and Quick Prime-3 perturbations were alternately performed followed by a single NPFE perturbation. Peak R at each dose was plotted against Mdel, fitted to a second order polynomial and the dose at which R was 200% (PC_200_) of the mean response to saline was interpolated.[68, 75, 81] Following *flexiVent*, mice were sacrificed by exsanguination (cardiac puncture and transection of the IVC) as per NYU IACUC approved protocol #s16-00447.

### Murine plasma, BAL and analyte assessment

Lungs were lavaged with 1cc-cold normal-saline and cytospin stained with H&E (Hema-3, Fisher).[82] Plasma was collected by 18g cardiac puncture in 1cc-syringe with 10μL-heparin (100units/mL). Specimens were aliquoted and stored at -80°C.

### Histology/Quantification of murine lung morphology

Lungs were fixed *in situ* with 4% paraformaldehyde (Sigma) at 25 cmH_2_O and stored in 70% ethanol (4°C). Lungs were processed through a series of graded ethanol, from 70% to 100%, then into Xylene, and finally infiltrated with paraffin (Leica Peloris tissue processor). Once embedded in paraffin blocks lungs were sectioned at 5 μm onto charged slides using a rotary microtome and stained with hematoxylin and eosin, as previously described.[49, 83] The optimal lung sampling has been discussed in several studies. [34, 84–86] To view a maximal amount of lung area, longitudinal coronal sections were cut on a plane to include mainstem bronchi.[49] The stained slides were then digitally scanned (Slidepath, Leica). Investigators were blinded to experimental condition during selection and measurement of all fields.[49] To select fields for analysis, a grid of squares (520 μm x 520 μm) was laid over the entire lung section in Slidepath (Leica) at 2X magnification. Squares/fields were chosen systematically by selecting every fifth field starting from top left to right of the grid to optimize unbiased random sampling of the section.[86] Squares/fields that were not entirely tissue, such as those at the edge of the lung, were excluded. This method was repeated until 10 fields were selected. Each field was cropped at 20X magnification and treated as a separate image for the purpose of area fraction and mean linear intercept quantification. Images were converted to 8-bit gray-scale, automated thresholding (ImageJ) was used to distinguish airspace and tissue and each image was binarized (Image J) for further analysis, S1A and S1D Fig. Area fraction was measured to express the biovolume-to-airspace ratio.[34, 87, 88]

#### Mean linear intercept (MLI)

The customary number of chords measured has varied from 300 measurements per lung to as high as 7000 measures per lung.[84, 87, 89, 90] On average 582 chords per image and 5820 chords per lung were assessed. Each binarized image was overlaid with 15 semi-transparent, horizontal test lines (opacity = 50%) spaced 35.4 μm apart (Adobe Photoshop). Discrete chords traversing alveolar septa, isolated based on pixel color, were measured and mean linear intercept was calculated (Adobe Photoshop, Image-J), S1E and S1G Fig.[84, 86, 89, 91, 92] This process was repeated for vertical test lines of the same spacing.[91] All chord lengths were measured using ImageJ.[84, 89, 92] Chord lengths were pooled per exposure group and analyzed as an average for each exposure group.

### Murine transcription factors

CREB, NF-κB, AKT, p70S6K, JNK, P38, ERK1/2 and STAT3/5 were measured in lung homogenates (#48-680/1MAG). Total β-tubulin (#46-713MAG) was used as a control. Mouse lung tissue lysates were assessed by SDS-PAGE; probed using Akt1/2/3 (H-136), p-Akt1/2/3 (Ser 473), RAGE (N-16) primary-antibodies and GAPDH (FL-335) (Santa Cruz-Biotechnology) as loading control.[93–95]

### Murine chemokines/cytokines

Human serum was assayed for sRAGE, CRP, and MMP-9 using Soluble Receptor, Cardiovascular, and Neurodegenerative multiplex assays (Millipore) and analyzed (200IS-Luminex). Murine BAL and plasma was assayed using #MCYTMAG-70K-PX32; EMD-Millipore, Billerica. Lysophosphatidic Acid (LPA) was quantified in human-serum and, murine-BAL and plasma by ELISA (Echelon-Biosciences).[11]

### Statistics

We identified biomarkers associated with the development of WTC-LI in the FDNY cohort using logistic regression adjusted for exposure, BMI at SPE and age at 9/11. Positive and negative likelihood ratios (LR+ and LR-) were calculated for each biomarker, S4 Table. Biomarker cut-points were determined to best optimize the models of association with WTC-LI as previously described.[12, 51–53] The continuous sRAGE data was log transformed due to a right-skew. In a logistic regression analysis using all available sRAGE data points (n = 279), continuous sRAGE (log transformed) was found to be positively correlated with development of WTC-LI (OR = 1.516, p = 0.560). After adjusting for BMI at SPE, age on 9/11 and exposure group, the OR of continuous log(sRAGE) was 1.983, p = 0.348. Both univariate and multivariate analysis of the continuous sRAGE showed positive association with the outcome. As often seen in biomarker studies, not the whole range of the continuous biomarker is informative to disease/outcome ascertainment. Thus, we dichotomized the biomarker using the Youden’s index which was calculated using all available data points (n = 279) as previously described. [96–98] Briefly, the Youden Index *(J) (J = Sensitivity + Specificity -1)* was utilized to identify a cutpoint to maximize sensitivity and specificity of the biomarker. The optimal cutpoint, 97 pg/mL, was then applied to all further analysis of cases and controls defined by the inclusion and exclusion criteria described in Fig 1.

*Flexiware*-7.5.4 (Scireq) was used for murine primary data acquisition and MasterPlex-QT (MiraiBio, Hitachi) for murine multiplex data. *SPSS*-23 (IBM) and *Prism* 6.07 (Graphpad) were used for data management and analysis of both human and murine data. Since there are documented phenotypic differences between WT and Ager^-/-^ mice at baseline, using ANOVA for multigroup comparison cannot differentiate the exposure difference in the context of genetic difference.[34] Therefore, similar to other recently published studies that have identified phenotypic differences, we have chosen to use compare the PM exposed to their controls within each genetic group, which is a primary interest of this study.[34] Comparisons were made by Student’s t-test or Mann-Whitney U depending on normal distribution of the data, Figs 2A–2D and 3B. Tissue resistance (R_tis_) across a range of frequencies was evaluated by multiple t-tests and corrected for multiple comparisons using the Holm-Sidak method, Fig 3A.

## Results

### FDNY WTC exposed cohort

#### Demographics

The baseline cohort (N = 801) was stratified based on BMI and FEV_1_ at MMTP entry. The study cohort was randomly selected from the baseline cohort after this stratification as previously described.[99] Additionally, only subjects with a full set of biomarkers required for this analysis were included in our final analysis (N = 185). In a comparison of major variables in the baseline cohort (N = 801) and study cohort (N = 185) we found no significant differences in FEV_1_% predicted, FVC% predicted and FEV_1_/FVC between cases and controls from each cohort. Additionally, BMI, age on 9/11 and exposure group did not differ between cohorts, S1 Table.

Subjects with WTC-LI (N = 67) and their controls (N = 118) did not differ by age at the time of 9/11, BMI at MMTP entry, racial composition or duration spent on the site during rescue/recovery efforts, Table 1. The number of months (median, IQR) that had lapsed from pre-9/11 spirometry exam to MMTP was also not different in cases 13 (7–20) and controls 13 (7–18); time from 9/11 to SPE evaluation and from MMTP entry to SPE also did not differ between cases and controls, Table 1. Cases had significantly higher BMI at SPE, Table 1.

**Table 1:** Clinical measures, biomarker prevalence and model definition.

Measure	Cases N = 67	Controls N = 118	OR (95%CI)*	
			Crude	Full Model
**Caucasian**	63(94.0%)	117(99.2%)	7.4(0.8–67.9)	
**Duration (months)**	2.0(1.0–4.5)	3.0(1.0–5.0)	0.9(0.8–1.1)	
**Age on 9/11**	41(36–46)	42(37–46)	1.0(0.95–1.1)	1.0(0.96–1.1)
**PFT at SPE**				
**FEV**_**1**_**% Pred**	72.3(66.5–74.5)	95.1(87.7–104.0)	Case Definition	
**FVC % Pred**	79.0(73.0–86.0)	98.0(92.8–105.0)	0.8(0.7–0.8)	
**FEV**_**1**_**/FVC**	71.2(64.7–77.1)	77.1(73.9–80.6)	0.9(0.8–0.9)	
**Months**				
**MMTP Entry-SPE**	29(16–49)	31(32–69)	0.99(0.98–1.0)	
**9/11-SPE**	48(28–64)	48(22–53)	0.99(0.98–1.0)	
**BMI**				
**MMTP Entry**	29.1(26.6–31.7)	27.9(26.2–30.5)	1.1(0.99–1.2)	
**SPE**	30.3(27.5–34.0)	29.0(26.5–31.2)	1.1(1.0–1.2)	1.1(0.99–1.2)
**Exposure**				
**Low**	13(19.4%)	14(11.9%)	Reference	Reference
**Intermediate**	36(53.7%)	83(70.3%)	0.9(0.3–2.5)	0.9(0.3–2.6)
**High**	18(26.9%)	21(17.8%)	0.5(0.2–1.1)	0.5(0.2–1.1)
Biomarker*				
**sRAGE**≥97 pg/mL	22(32.8%)	22(18.6%)	2.3(1.1–4.7)	2.2(1.1–4.7)
**CRP**≥2.4 mg/L	59(88.1%)	83(70.3%)	2.7(1.1–6.5)	2.8(1.2–6.9)
**MMP-9**<397 pg/mL	48(71.6%)	66(55.9%)	2.0(1.0–3.8)	2.0(1.0–4.0)
**IFN-**γ<8 pg/mL	41(61.2%)	53(56.4%)	1.9(1.0–3.5)	
**LPA**≥35 μM	17(25.4%)	16(13.6%)	2.5(1.1–5.5)	
**Full Model ROC**_**AUC**_: 0.72(0.65–0.80)				

Values are represented as Median(IQR), N(%), or OR (95% CI) as indicated.

*Logistic Regression Models for biomarkers adjusted for age on 9/11, Exposure, and BMI at SPE.

**Abbreviations: PFT**-Pulmonary Function Test; **FEV**_**1**_- Forced Expiratory Volume in 1 second; **FVC**-Forced Vital Capacity; **MMTP**-Medical Monitoring and Treatment Program; **SPE**-Subspecialty Pulmonary Exam; **BMI**-Body Mass Index; **sRAGE**-soluble Receptor for Advanced Glycation End-Products; **CRP**-C-reactive protein; **MMP**- Matrix Metalloproteinases; **OR**-Odds Ratio; **CI**-Confidence Interval; **IFN**-Interferon; **LPA**-Lysophosphatidic Acid; **ROC**-Receiver Operator Characteristic; **AUC**-Area Under the Curve; **mg**-milligram; **pg**-picogram; **mL**-milliliter; **μM**-micromolar.

#### Lung function and exposure at the WTC site

Exposure intensity, as defined by time of arrival at the WTC site, did not differ between cases and controls. Cases had significantly lower FEV_1_, FVC and FEV_1_/FVC than controls at SPE, Table 1. We also compared FEV_1_ at pre-9/11 to that measured at MMTP and found that the loss of FEV_1_ was no different in cases and controls between these two time points, with median (IQR) loss of 9.0% (5.0%-15.0%) for cases and 9.5% (3.0%-16.0%) for controls (p = 0.97). Loss of lung function was significant when we compared pre-9/11 FEV_1_ to that measured at SPE; median (IQR) for cases 17.9%(8.5%-28.1%) and 5.6%(0.2–12.2%) for controls.

#### sRAGE levels obtained soon after exposure are associated with the development of WTC-LI in the FDNY cohort

All univariate and multivariate models were adjusted for potential confounders—age on 9/11, BMI at SPE, and exposure intensity as defined by time of arrival at the WTC site. Inflammatory markers such as CRP have been shown to increase with age and FEV is known to decline with age. [4, 5] BMI is known to be associated with decreased lung function and is significantly different between cases and controls at SPE. The lung function measurements obtained at SPE provided the basis of our case definition and so we also adjusted for BMI, a potential confounder. [6] Finally, our group has previously published that there is a significant exposure intensity response gradient in the loss of FEV_1_ in the full cohort, therefore we have also adjusted for exposure group in our models. [7]

Biomarkers with relevance to the RAGE signaling pathway were assessed for their association with the development of WTC-LI. Those that were found to be significantly associated with the development of WTC-LI were included in Table 1. Crude ORs were assessed and sRAGE ≥ 97pg/mL, CRP ≥ 2.4mg/L, MMP-9 < 397pg/mL, IFN-γ < 8pg/mL, and LPA ≥ 35μM increased the odds of developing WTC-LI by 130%, 170%, 100%, 90% and 150% respectively, Table 1.In the full model, multivariate logistic regression included sRAGE≥ 97pg/mL, CRP≥ 2.4mg/L, and MMP-9< 397pg/mL and was adjusted for age on 9/11, BMI at SPE and exposure intensity. IFN-γ and LPA were no longer significant predictors. sRAGE≥ 97pg/mL, CRP≥ 2.4mg/L, and MMP-9< 397pg/mL were found to increase the odds of developing WTC-LI by 120%, 180%, and 100%, respectively, and had acceptable association (ROC_AUC_ of 0.72), Table 1. Additionally, we assessed the LR+, LR- and ROC_AUC_(95%CI) of each of the confounder adjusted final model biomarkers and the overall full model, S4 Table. Our positive likelihood ratios represent small increases in probability of morbidity given a positive test. The ROC_AUC_ of each of the contributing biomarkers also represented less of an association than the full model, S4 Table.

Since the timing of lung function assessment is a potential confounder, we included time elapsed between MMTP-Entry and SPE, and between 9/11 and SPE in our regression analyses. Time, as measured by months from 9/11 to SPE and MMTP entry to SPE, was insignificant in all models and did not significantly affect other covariates, Table 1. The Interaction between BMI and age was also explored. We found that there was no significant interaction between BMI and age, OR (95% CI); 1.011(0.998–10.24).

### Murine PM Model

#### Mice lacking RAGE are protected against loss of FEV

To determine the importance of RAGE, we compared+ WT and Ager^-/-^ mice in a model of PM aspiration. WT mice evaluated one day after PM exposure had significantly reduced FEV_0.2_, Compliance (C), and increased respiratory resistance (R) compared to controls, Fig 2A–2C. FEV was additionally measured at 0.05 and 0.1 seconds; FEV_0.1_ was significantly decreased in WT-PM mice compared to their PBS controls, S3 Table. Similar differences for baseline respiratory mechanics were seen in elastance, tissue elastance (H), Quasi-static Compliance (C_st_), and Parameter-A (A); however, in mice devoid of Ager, none of the above parameters were significantly different after PM exposure, Fig 2 and S3 Table.

#### Ager^-/-^ protects mice from PM airway hyperreactivity

Since airway reactivity is a prevalent finding in the WTC-exposed population, we explored reactivity. PC_200_, the provocative concentration required to double R from baseline, was interpolated.[68, 81, 100] PM-exposed WT mice required a significantly lower concentration of methacholine (Mean ± SEM: 76.63 ± 15.36 mg/mL) to produce response compared to PBS controls (174.6 ± 43.93 mg/mL), Fig 2D. Ager^-/-^ mice exposed to WTC-PM did not differ significantly in hyperreactivity from their PBS controls, Fig 2D.

#### Changes in murine respiratory mechanics after PM exposure occur predominantly in the peripheral airways

To separate the effects of particulate exposure on central and peripheral airways, tissue resistance was derived at frequencies between 1 and 20.5Hz from raw input impedance data collected during baseline mechanics, Fig 3A. Lower frequencies are associated with smaller caliber airways. Mean tissue resistance (R_tis_) of WT-PM mice was *higher* at each frequency compared to WT-PBS, whereas Ager^-/—^PM mice had *lower* R_tis_ compared to their PBS controls. R_tis_ was significantly different after PM exposure in both WT and Ager^-/-^ mice at 1 Hz indicating obstruction in the peripheral airways. At higher frequencies, R_tis_ was not significantly different between any of the exposure groups. Tissue Damping (G), a reflection of the viscoelasticity and resistance in the alveoli/small airways, was significantly higher in WT-PM mice than in controls, whereas Ager^-/-^ mice exposed to PM were protected, Fig 3B.

#### Ager^-/-^ protects mice from histologic changes

**Fig 4:**
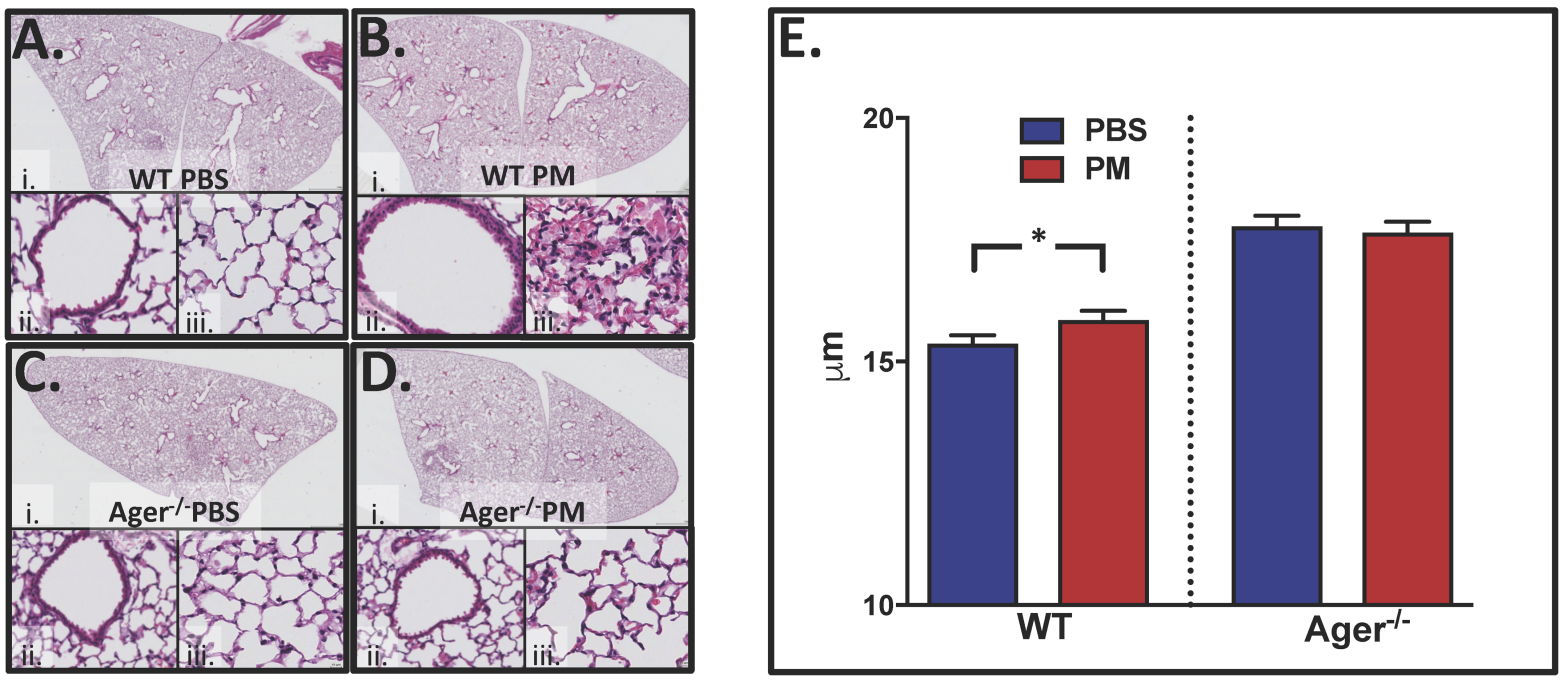
Quantifiable changes to lung histology after PM exposure. Light microscopic examination of representative hematoxylin and eosin stained sections of lung tissue 24 hours after exposure. (i) Images at 2X while, (ii, iii) are at 40X magnification. (A) WT-PBS exposed mice had normal lung architecture and no infiltrates, whereas (B) WT-PM exposure led to infiltrates, focal acute bronchoalveolar inflammation and interstitial thickening. (C) Ager^-/-^ mice that aspirated PBS and (D) WTC-PM showed no remarkable changes to normal lung architecture. (E) MLI was significantly (*) higher after PM exposure in WT mice but there was no change in Ager^-/-^ mice after PM exposure compared to their controls.

WT mice had normal lung architecture and no infiltrates after PBS, Fig 4A. WTC-exposed mice after 24-hours had infiltrates, focal acute bronchoalveolar inflammation and interstitial thickening, Fig 4B. WT and Ager^-/-^ showed no remarkable changes in area fraction compared controls, Fig 4A–4D. Median (IQR) area fraction was 28.2(24.7–31.5) for WT-PBS, 30.9(25.6–35.3) for WT-PM, 28.2(24.1–30.2) for Ager^-/—^PBS and 27.2(21.8–29.7) for Ager^-/—^PM. Using the same images, we evaluated mean linear intercept (MLI), which is the mean free distance of gas exchange surfaces within the acinar surface complex, Fig 4E. WT mice exposed to PM had significantly increased MLI compared to WT-PBS controls. Ager^-/-^ mice were protected from changes to MLI after PM exposure when compared to their PBS controls.

#### Ager^-/-^ mice show differential expression of chemokines/cytokines in BAL and plasma after PM-aspiration

**Fig 5:**
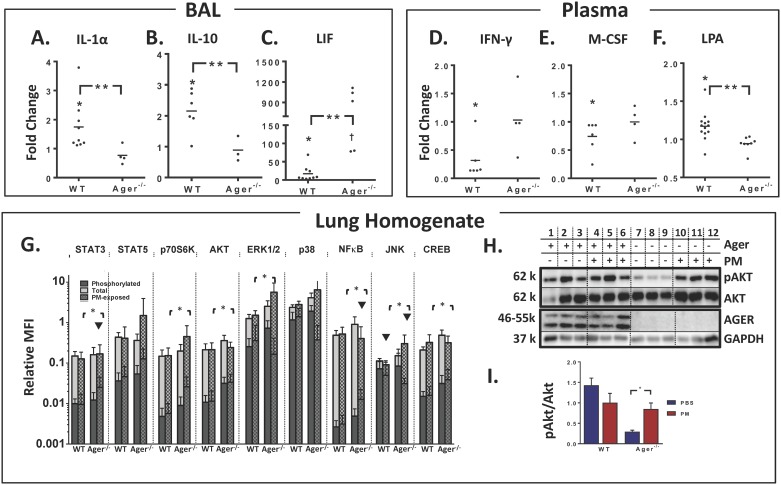
**Cytokines and transcription factors of murine particulate model**, biomarker profile in BAL (A-C) and plasma (D-F) expressed as fold change of PM-exposed WT and Ager ^-/-^ mice over their respective PBS controls. In BAL, IL-1α (A) and IL-10 (B), had significantly elevated fold change in WT-PM compared to PBS controls (*). Additionally, WT-PM expressed significantly higher fold-change in IL-1α (A) and IL-10 (B) compared to Ager ^-/—^PM. Ager ^-/—^PM expressed significantly higher fold-change in LIF (C) compared to WT-PM (**). LIF expression was also significantly higher in both WT-PM compared to WT-PBS and Ager^-/—^PM compared to Ager^*-/—*^PBS (†). In plasma comparing WT-PM and WT-PBS, IFN-γ (D) and M-CSF (E) were significantly lower, while LPA was higher (F), (*). Additionally, LPA fold-change was lower in Ager^-/—^PM compared to WT-PM (**). (G) Phosphorylated and Total Levels of Transcription Factors, expressed as MFI relative to β-tubulin. Phosphorylated levels of transcription factors are superimposed in a darker color over the total. Expression of WT is shown in the first two columns of each subdivision, followed by Ager^-/-^ mice. PM exposure is shown with hash marks. Ratio of Phosphorylated/total protein was significant between WT-PM and Ager^-/—^PM for transcription factors denoted by (*). Ratios significantly different between PM and PBS control are shown by (▼). (H) Western blot of lung homogenates was probed for phosphorylated AKT (pAKT), total AKT (AKT), AGER, and GAPDH as a protein-loading control. Representative images shown; n = 3 for each condition. Relative Phosphorylation of AKT is shown in panel (I) pAKT/total AKT was derived from the primary blots, n = 3 for each condition, mean ± SD. Ager^-/-^ exposed to PM had significantly greater pAKT/AKT compared to PBS controls, (*).

Median and interquartile range (IQR) of analytes in BAL/plasma are included in S5 and S6 Tables. Preliminary assessment of chemokines/cytokines revealed insignificant baseline differences between WT-PBS and Ager^-/—^PBS in all but one serum analyte, S5 and S6 Tables. **i. BAL** *of* WT and Ager^-/-^ mice both had macrophages >90% for all PBS-exposed mice, while PM caused significant neutrophilia, S3 Table. The fold-change expression of chemokines/cytokines after PM exposure was quantified for WT-PM and Ager^-/—^PM compared to their respective PBS controls. WT and Ager^-/-^ mice exposed to PM both expressed significantly elevated pro-inflammatory G-CSF, IL-6, LIF (Fig 5C), KC, MIP-1α/1β, M-CSF, MIG, and VEGF. WT mice additionally expressed higher fold-change IL-(1α, 5, 9, 10) (Fig 5A and 5B), MCP-1, and MIP-2, whereas Ager^-/—^PM mice expressed higher IL-2 compared to their respective PBS controls. Ager^-/-^ mice exposed to PM displayed significantly lower IL-1α and IL-10, and significantly higher LIF when compared to WT-PM, Fig 5A–5C.

**ii. Plasma** reflected generally higher concentrations of chemokines/cytokines compared to BAL, but fold-change of analytes in WT mice after PM exposure was lower in many pro-inflammatory cytokines. WT-PM and Ager^-/—^PM had decreased fold-change expression of GM-CSF, IL-17, MIP-2, and RANTES compared to their PBS controls. WT-PM had additionally lower fold-change expression of IL-2, IL-12(p40), IFN-**γ (**Fig 5D) and a trend to lower M-CSF, Fig 5E. Ager^-/—^PM had lower levels of IL-7, IL-12 (p70), MCP-1, VEGF, and higher levels of MIG compared to PBS. WT-PM had increased fold-change of plasma LPA; Ager^-/-^ mice did not demonstrate any change in plasma LPA after PM exposure, and also expressed significantly lower LPA compared to WT-PM, Fig 5F.

#### Lung homogenates from WT and Ager^-/-^ mice show differential expression of protein kinases after PM exposure

**i. Multiplex**: Ager^-/—^PM had significantly higher expression of phosphorylated CREB, NFкB, AKT, and p70S6K compared to WT-PM. Phosphorylated p38 and ERK1/2 were lower in concentration in Ager^-/—^PM homogenates compared to *Ager*^-/—^PBS and WT-PM. Total ERK1/2 and p38 protein were higher in Ager^-/—^PM compared to WT-PM.

When comparing the ratios of phosphorylated/total protein, Ager^-/—^PM expressed higher CREB, NFкB AKT, p70S6K, and STAT3 compared to WT-PM. PM did not induce any of the phosphorylated or total transcription factors in WT compared to controls. PM exposure decreased phosphorylated/total JNK in both WT and Ager^-/-^ mice; however, WT-PM ratio was significantly higher compared to Ager^-/—^PM for JNK and ERK1/2, Fig 5G.

**ii. Immunoblots**: RAGE was similarly expressed in WT in both PBS and PM exposed mice (Fig 5H, Lane 1–6). Ager^-/-^ mice did not express RAGE (Fig 5H, Lane 7–12). Similar to the results from the bead-based assay, the pAKT/AKT ratio was not significantly different between WT-PBS and WT-PM, Fig 5I; however, in contrast to the bead-based assay, Ager^-/—^PM had significantly increased pAKT/AKT ratio compared to Ager^-/—^PBS (p = 0.017), Fig 5I.

## Discussion

Mediators of metabolism have been associated with WTC-LI in previous studies of the FDNY cohort. [10, 51, 52] RAGE, a biologically plausible mediator of PM-induced lung disease, was the focus of this investigation. In the final multivariate model, elevated sRAGE and CRP, and decreased MMP-9, were associated with developing WTC-LI. CRP is a highly sensitive marker of acute inflammation and/or tissue damage and levels ≥2.4mg/mL have been associated with doubling the relative risk of a coronary event.[101] MMP-9 cleaves membrane-bound RAGE shedding sRAGE.[18] Studies investigating the role of sRAGE in different disease states including sepsis, lung disease, diabetes and cardiovascular disease often show conflicting information with regard to the directionality of sRAGE expression and severity of disease.[22, 23, 102–105] The inverse relationship between sRAGE and MMP-9 seen in the WTC-exposed FDNY cohort maintains plausibility because MMP-9 levels may decrease as it is consumed to cleave RAGE. In our previously published multivariate models, MMP-9 was not associated with development of WTC-LI in the FDNY cohort.[106] There are likely several contributors to this finding. Although both studies utilized commercially available MMP-9 assays, the manufacturer and the analytes included in the multiplexes differed and therefore different antibodies, sample dilution requirements, and antibody cocktails may have played a role.[107] In addition, the significance of the association of MMP-9 with the development of WTC-LI was assessed in multivariate models that had differing analyte components in these two papers.

Our murine model of PM-induced inflammatory lung disease allowed us to study changes in lung function seen soon after PM exposure.[5, 49] Lung function measures have been shown to be of clinical relevance in the setting of acute exposures.[5, 6, 38, 49, 50, 108–110] In a murine model of fibrosis, mice received a single intratracheal dose of silica which induced fibrosis at the 14 day time point.[38] In pediatric studies, increasing concentration of air pollutants and particulate counts were associated with decline in spirometric measurements within 24 hours.[108–110] Elevation of ambient PM exposure was associated with reduced pulmonary function measurements of adults in Framingham Heart study assessed on the next day.[111] Both the FDNY-WTC cohort and WT-PM exposed mice show airway hyperreactivity and a decrease in FEV after an acute PM exposure. In the FDNY cohort intense early exposure to particulate matter on 9/11 and in the weeks following 9/11 during clean-up efforts is a significant predictor of later lung function decline, while in the mice a single high intensity exposure results in quantifiable changes to lung function at the 24 hour time point. Similarities were also seen in analysis of the human induced sputum and murine BAL. In the FDNY-WTC cohort, induced sputum showed neutrophil predominance that correlated with greater exposure intensity, whereas murine BAL showed neutrophil recruitment in both WT and Ager^-/-^ after PM exposure.[5] This murine data is concordant with prior murine data showing neutrophil predominance, decrease in macrophage content and hyperresponsiveness 24 hours after PM exposure.[49]

Mice lacking RAGE were not protected from PM-induced neutrophilia, but were protected from both loss of FEV and compliance. Furthermore, these mice did not develop an increase in airway resistance and reactivity after PM exposure compared to their controls. This suggests that RAGE may act in collaboration with other mediators leading to eventual lung injury. We also noted that Ager^-/—^PBS exposed mice responded to a significantly lower dose of methacholine compared to WT-PBS exposed mice. This may be because R was lower at baseline for the Ager^-/-^ mice, thus requiring less methacholine to double the resistance. Inherent phenotypic variations between WT and Ager-/- mice have been observed elsewhere [34, 112]. We observed baseline differences in MLI between WT-PBS and Ager^-/—^PBS. Together these data suggest the architecture of the lungs in Ager^-/-^ mice are altered in a way that affects baseline lung function. For this reason, comparisons in this study were limited to comparisons of background matched controls.

WTC-exposed cohorts developed lower respiratory symptoms after 9/11 and forced oscillometry measurements in these subjects showed that the disease process occurs in the small airways.[113–115] Similarly, our murine data support the finding that small airways are more affected than large caliber airways after acute exposure to PM, which may help explain the heterogeneous pattern of lung disease observed on histology. Analysis of R_tis_ indicated that WTC-PM exposure is associated with smaller airways obstruction. These data correlate with the histology of the murine lungs. WT-PM showed heterogeneous changes to lung architecture, which was indicated by lack of significant findings in area fraction, but significantly increased MLI, while Ager^-/-^ mice showed no difference in either measurement. MLI is a function of lung volume. [116, 117] The increase in MLI observed in WT-PM compared to their PBS controls can either be from destruction of alveolar septation or lung distension, however the volume of lung is required to differentiate them.[118] Unfortunately, current *flexiVent* technology is unable to provide us the residual lung volume. The localized inflammation observed on histology may also be due to the time point examined. Since the scope of this experiment was to understand acute changes that occur after WTC-PM exposure, we focused on measurements after 24-hours.

Many chemokines/cytokines were involved in the inflammatory process in both WT and Ager^-/-^ mice after exposure to PM. We highlighted those analytes with a significant fold induction in WT expression after PM exposure compared to PBS controls. In our prior *in vitro* work, alveolar macrophages from normal human BAL samples express higher levels of IL-1α and IL-10 24 hours after exposure to WTC-PM.[48] These analytes were also elevated in the BAL of WT-PM, but not in the Ager^-/-^ after PM exposure. Interestingly, although WT-PM expressed an elevation in both IL-1α and IL-10, IL-1α’s function is primarily pro-inflammatory, whereas IL-10 is anti-inflammatory. Data on IL-1α in the setting of pulmonary inflammation are mostly centered on early neutrophil recruitment in response to an infectious challenge and necrotic cell death.[119] IL-10 is usually involved in the anti-inflammatory signaling pathway, and promotes long-term immunity through memory CD8+T-cells.[120] Ager^-/—^PM expressed higher fold change in LIF compared to WT-PM. LIF is a cytokine from the IL-6 family that has been shown to promote traditionally inflammatory biological activities including cell proliferation and survival.[121, 122] However, recent studies also show its anti-inflammatory properties in the lung and other organs.[123–125]

All three BAL analytes of interest—IL-1α, IL-10 and LIF—induce STAT3 signaling. Despite having increased IL-1α, IL-10 and LIF after PM-exposure, WT mice did not show an induction in STAT3 at 24 hours. In contrast, Ager^-/-^ mice did not have increased IL-1α or IL-10, but did have increased LIF after PM exposure and showed a significant increase in phosphorylated STAT3. Previous studies showed that binding of LIF to LIF-receptor-gp130 heterodimer leads to the phosphorylation of STAT3 by JAK.[126] Additionally, LIF has been shown to have tissue-protective effects in murine models of pneumonia, and the presence of STAT3 in alveolar epithelial cells has been shown to have protective effects in inflammatory lung injury.[127] Thus, it is possible that the LIF-STAT3 pathway is a key mediator, and WT-PM’s 17-fold increase in LIF was insufficient to induce STAT3 activation, whereas the Ager^-/—^PM had a 646-fold increase and was positively associated with STAT3 phosphorylation. It is also possible that the activation and phosphorylation of STAT3 was not fully captured at 24h.

In plasma, many pro-inflammatory cytokines were lower in WT-PM compared to PBS. WT-PM mice had decreased IFN-γ and increased LPA; similarly, firefighters with WTC-LI were more likely to have IFN-γ<8pg/mL and LPA≥35μM. WT-PM mice additionally had decreased M-CSF. Although IFN-γ and M-CSF are both pro-inflammatory cytokines involved with monocyte activation and induce STAT, mice deficient in RAGE did not show dampening of these cytokines compared to their PBS controls and did not have a universal dampening of pro-inflammatory cytokines after PM exposure. This may indicate that there a balance of pro- and anti-inflammatory cytokines after PM exposure determine downstream effects on lung injury.

We observed that LPA, a known ligand of RAGE was increased in the plasma of WT-PM exposed mice. Ager^-/-^ mice exposed to PM were protected from both lung function loss and elevations in LPA. Lower levels of LPA, a component of the cell membrane, are found in Ager^-/-^ mice. This may be reflective of less cell membrane disruption in the Ager^-/-^ mice that are PM exposed compared to WT. This suggests that RAGE may play a central role in the development of pulmonary dysfunction after high intensity environmental exposures.

RAGE is expressed in many cell types including alveolar type epithelium, monocytes/macrophages, granulocytes and T cells and has been suggested to play a role in both innate and adaptive immunity.[18, 21, 24, 25] Since the exposure to PM is known to cause a systemic response and many of these cell types are key mediators in the lung, we investigated biologically plausible mediators of RAGE signaling to better understand the signaling mediators and relevant pathways involved in PM-induced lung injury. The MAP kinases p38 and JNK are activated upon macrophage activation, a key cell type in the lung expressing RAGE.[128] Additionally, RAGE is expressed by granulocytes and involved in their adhesion and migration. Specifically, activated neutrophils display enhanced PI3 kinase-dependent signaling and RAGE-dependent binding to AGE collagen.[129] Additional intermediates involved in RAGE signaling include AKT, JAK/STATs and NF-кB.[130, 131]

While RAGE has been shown to be altered in certain disease states, we observed similar expression of RAGE in lung tissue of both PM and PBS exposed WT mice, suggesting that PM exposure did not directly alter the expression of cell bound RAGE. Future work evaluating levels of Ager-mRNA may further clarify PM-effect. Despite better function and pathological endpoints, Ager^-/-^ mice after PM exposure have greater induction of the phosphorylated protein kinases. Ager^-/-^ murine lungs exposed to PM include significantly higher expression of phosphorylated CREB, NFкB, AKT, and p70S6K compared to WT-PM; however, phosphorylated p38 and ERK1/2 were lower in concentration in Ager^-/—^PM compared to both Ager^-/—^PBS and WT-PM. Ager^-/-^ mice exposed to PM had significantly higher expression of total ERK1/2 and p38 protein compared to WT-PM. Of interest, pAkt/Akt as quantified in by multiplex showed a significant difference between WT-PM and Ager^-/—^PM that was not observed in the immunoblots. Additionally, in the immunoblots we show that the pAKT/AKT ratio was significantly higher in Ager^-/—^PM than in their PBS-controls while WT showed no significant difference in pAKT/AKT ratio after PM exposure. These conflicting data may be the result of the differing antibodies used in each assay.[107]

RAGE has been the focus of targeted therapeutic trials. Modulators of RAGE have been studied in regards to several chronic states such as diabetes, cancer, amyloidosis, neurodegenerative diseases such as Alzheimer’s type dementia and even aging.[16, 20, 132, 133] The decoy receptor abilities of sRAGE have been studied extensively in many of these conditions. In murine diabetes models chronic administration of sRAGE protects against many end-organ complications but does not normalize hyperglycemia or dyslipidemia. This has suggested that other RAGE antagonists may be suitable to investigate as adjunctive therapies.[16, 20]

In Alzheimer’s type dementia RAGE has been shown to bind amyloid-β (Aβ) mediating the toxic effects of Aβ oligomers in neurons. Preclinical studies of PF-04494700, an oral RAGE inhibitor, decreased brain Aβ load in transgenic mice and improved their performance on behavioral assays.[134] Furthermore, a soluble fusion protein inhibitor of RAGE signaling decreased soluble brain amyloid beta levels, decreased plaque load, reduced inflammatory cytokine levels and improved measures of behavior in a murine model of Alzheimer’s type dementia.[135]

RAGE expression can also be down regulated by peroxisome proliferator-activated receptor-γ (PPARγ agonists.[136, 137] Several groups have identified the therapeutic potential of PPARγ in COPD.[138–140] PPARγ is a nuclear hormone receptor and is involved in adipocyte differentiation and macrophage activation [141] In a recent case-control study of subjects with COPD, SNPs of PPARγ were associated with the development of COPD.[142] Furthermore, genetic polymorphism of PPAR*γ* have been linked to the development of asthma.[140] Finally, more recently some RAGE inhibitors have focused on the interaction of the cytoplasmic tail of RAGE (ctRAGE) and intracellular effector mammalian diaphanous 1 (DIAPH1). After screening 58,000 small molecules, 13 were identified as competitive inhibitors of ctRAGE and DIAPH1. These are potentially bioactive therapeutic agents that may be suitable agents for future *in vitro* and *in vivo* investigations.[16, 20, 143]

There are several limitations to our study. We have not chosen to assess the effects of RAGE as it pertains to a given cell type. In the lung RAGE is most highly expressed in alveolar epithelial type 1 cells and has additionally been shown to be expressed in many cell types including alveolar epithelial type 2 cells, vascular endothelial cells, alveolar macrophages and the smooth muscle cells of the airways.[24, 25] Our results show that the effects of RAGE are likely due to its expression by multiple cell types. Specifically, the cytokines/chemokines elaborated following PM exposure in this investigation and our prior *in vitro* work have several different parent cells and can affect several cell types. Currently the role of RAGE is being extensively studied in a series of loss and gain of function experiments. Over-expression of RAGE in alveolar epithelium is associated with airspace enlargement, increased apoptosis, increased MMP-9 expression, decreased elastin expression, alveolar hypoplasia and led to impaired endothelial cell differentiation.[144, 145] RAGE also has multiple ligands which contribute to its importance in both acute and chronic disease. Due to its pleotropic effects, varied cellular expression and the fact that PM exposure leads to a systemic response we have chosen to utilize a RAGE deficient murine model to assess the importance of RAGE in PM exposure, similar to the experimental designs of several other studies.

Similar to other groups, in our human study we have assessed serum levels of sRAGE as a minimally invasive biomarker of OAD. While it is true that our LR+ represent small increases in probability of morbidity, there are several possible contributors to this observation. Metabolic syndrome is prevalent both in our society and our cohort, yet metabolic derangement is a very heterogeneous process. When not controlled for, this heterogeneity obscures important differences in analyte expression, confounding likelihood ratios. Also notable, the AUC values of the single predictors do not differ substantially from the AUC of the full model, therefore the relative predictive power of sRAGE, CRP and MMP-9 cannot be established at this time. Furthermore, our biomarkers only represent a small subset of the bioactive analytes that can be found circulating in the affected human. Therefore, future work will include assessment of the metabolome to further optimize our predictive models.

Dichotomization of continuous variables is a common practice in biomarker research, however it can be a source of bias and decreased statistical power, and lead to the misclassification of subjects. Further validation of sRAGE as a biomarker must be done in an external population before its clinical applicability can be established. Our future work will validate the utility of serum sRAGE as a biomarker of WTC-LI in the larger FDNY cohort, which also includes smokers. Additionally, validation of RAGE as a biomarker of PM associated lung disease will need to occur in other cohorts.

Our work suggests that RAGE-associated inflammation may play a role in PM-induced lung disease, but the precise underlying mechanisms remain to be elucidated at this point. In the human WTC cohort, persistent FEV_1_ loss and the development of methacholine responsiveness have been observed. Future work will attempt to translate these findings in a long-term murine model of PM exposure. This may allow us to further evaluate the mechanisms underlying both the acute and chronic changes observed in mice after PM exposure. Finally, WTC-PM from five sites was used to simulate the real-world exposure of firefighters who, in the context of their rescue and recovery efforts, were not bound to one specific location or PM size; however, it is beyond the scope of our work to identify specific PM components that may be responsible.

In conclusion, this is the first study that investigates RAGE as a biomarker of PM-induced lung injury. We demonstrate that sRAGE is a biomarker of WTC-LI in the FDNY cohort and show that WTC-PM exposure causes inflammation and loss of lung function in a murine model. Finally, we show evidence that loss of RAGE is protective against murine lung injury seen within a day of PM exposure suggesting a potential therapeutic target for PM-induced lung disease.

## Supporting information

S1 Table Comparison of baseline and study cohorts. (TIFF)

S2 Table Analysis of World Trade Center particulate matter by x-ray fluorescence. (TIFF)

S3 Table Measures of lung function and BAL differentials. (TIFF)

S4 Table Likelihood ratios and ROC_AUC_ by biomarker. (TIFF)

S5 Table BAL cytokine/chemokine profile. (TIFF)

S6 Table Plasma cytokine/chemokine profile. (TIFF)

S1 Fig Quantification of lung histology and overview of image analysis. A. Select Fields. B. Converted to Grayscale (8-bit) C. Threshold Adjusted D. and binarized. E. Image was overlaid with 15 semi-transparent, horizontal test lines F. Discrete cords were isolated based on pixel color. This process was repeated for vertical test lines of the same spacing G. Chord lengths were measured.
(TIFF)
