# Supplementary figures and images for: Receptor for advanced glycation end-products and World Trade Center particulate induced lung function loss: A case-cohort study and murine model of acute particulate exposure
Source: PLoS One. 2017 Sep 19;12(9):e0184331. doi: 10.1371/journal.pone.0184331 (PMC5604982; doi:10.1371/journal.pone.0184331)

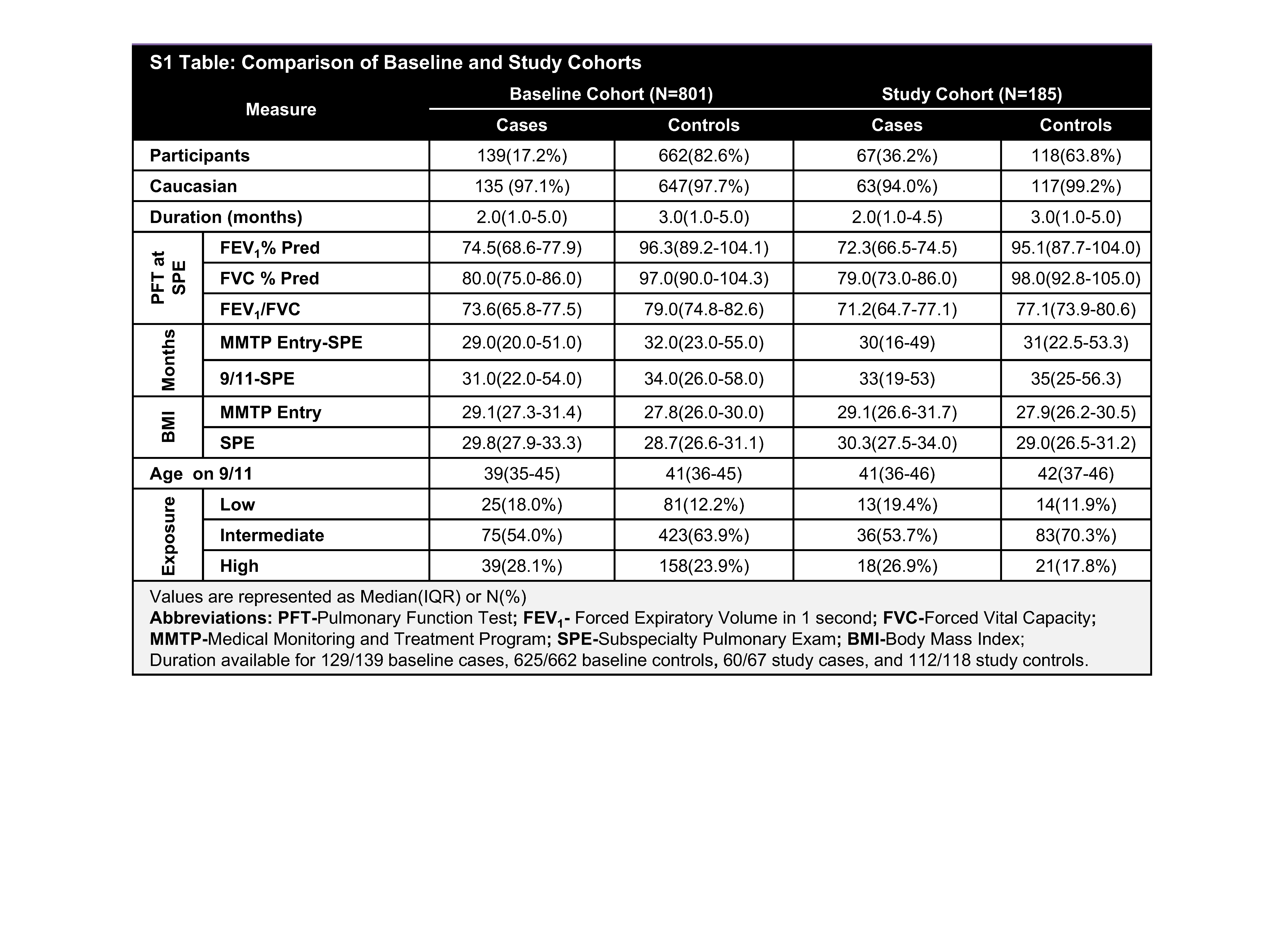

Supplement: S1 Table — (TIFF) [file pone.0184331.s001.tiff]

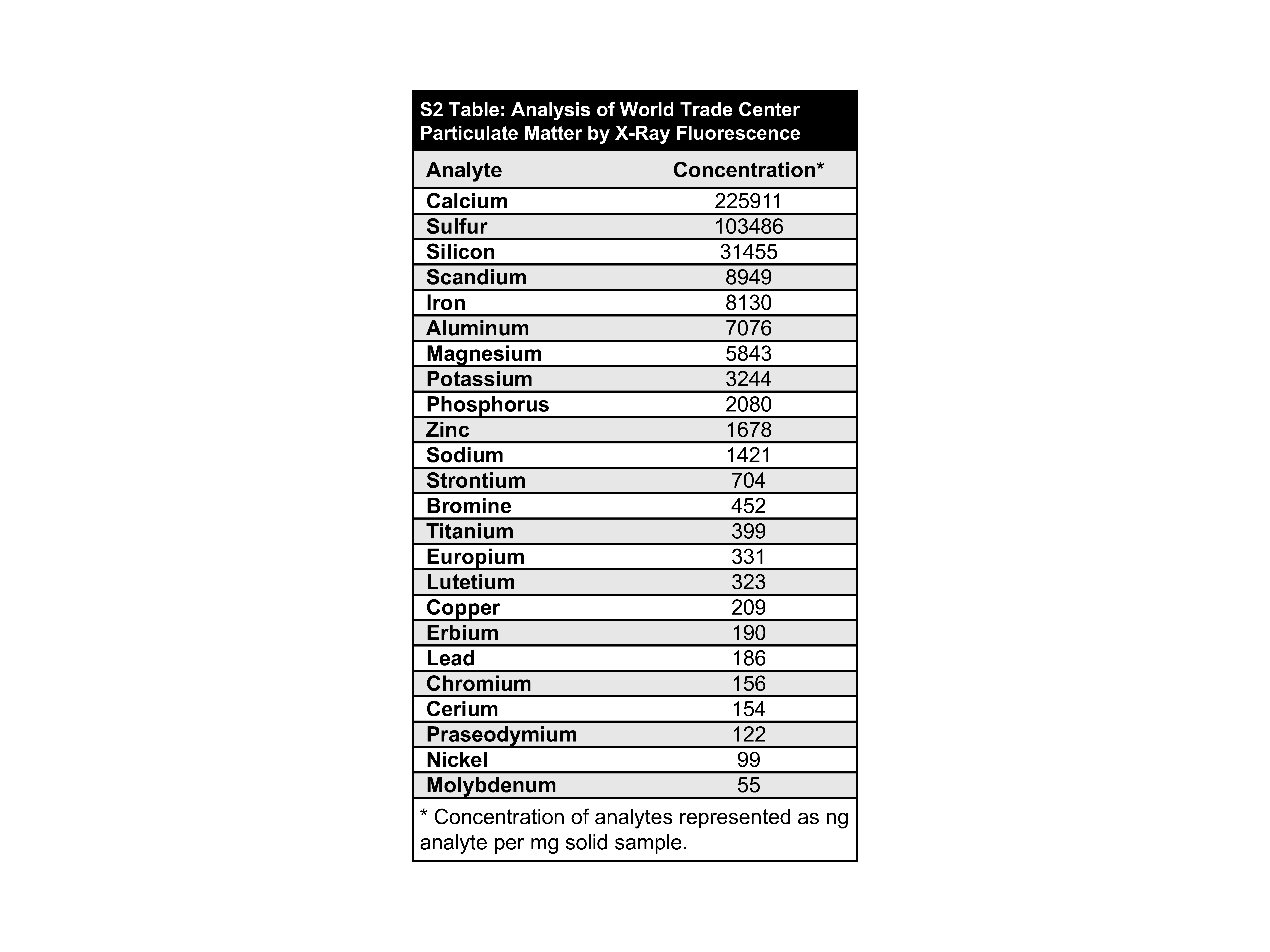

Supplement: S2 Table — (TIFF) [file pone.0184331.s002.tiff]

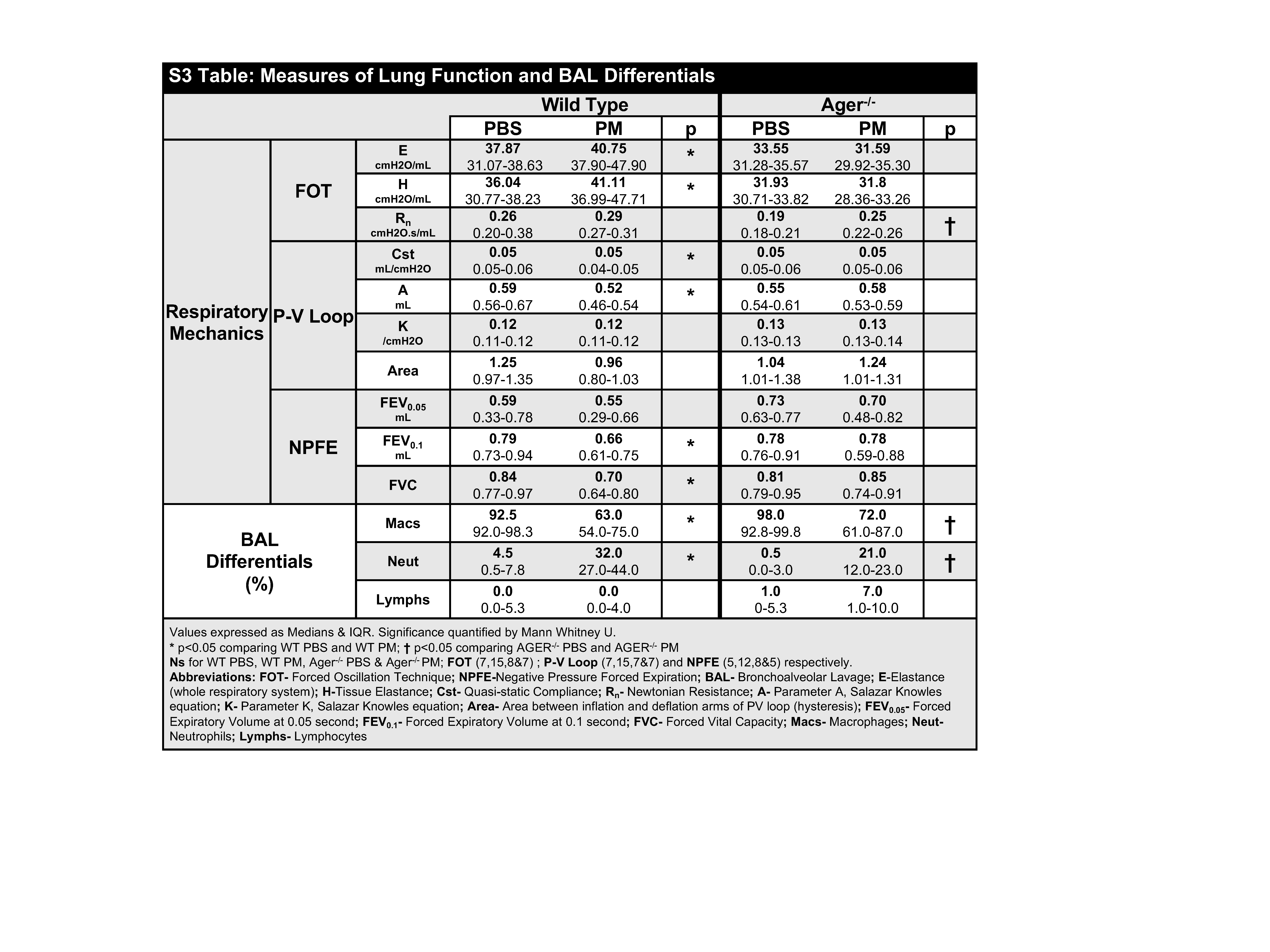

Supplement: S3 Table — (TIFF) [file pone.0184331.s003.tiff]

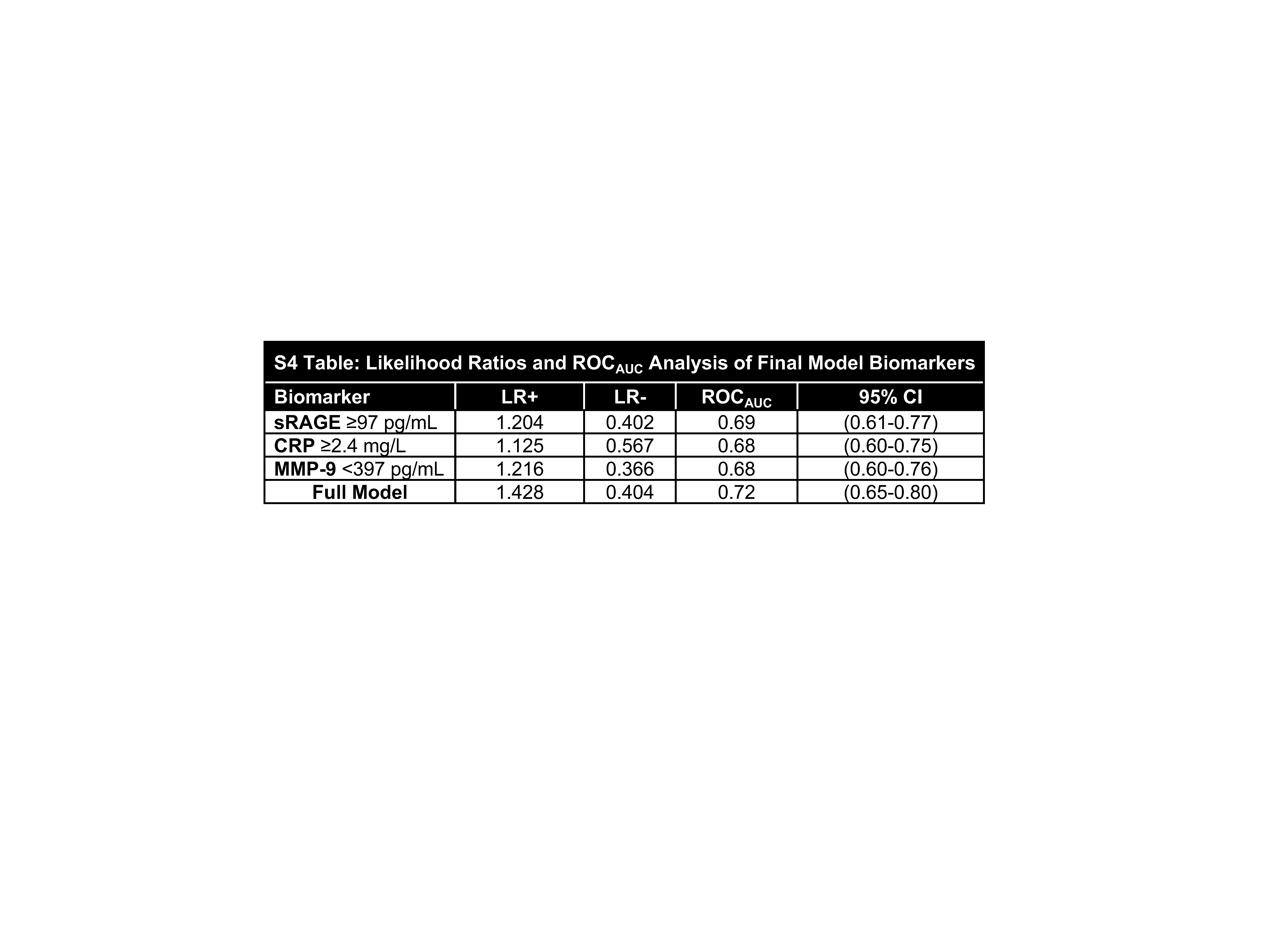

Supplement: S4 Table — (TIFF) [file pone.0184331.s004.tiff]

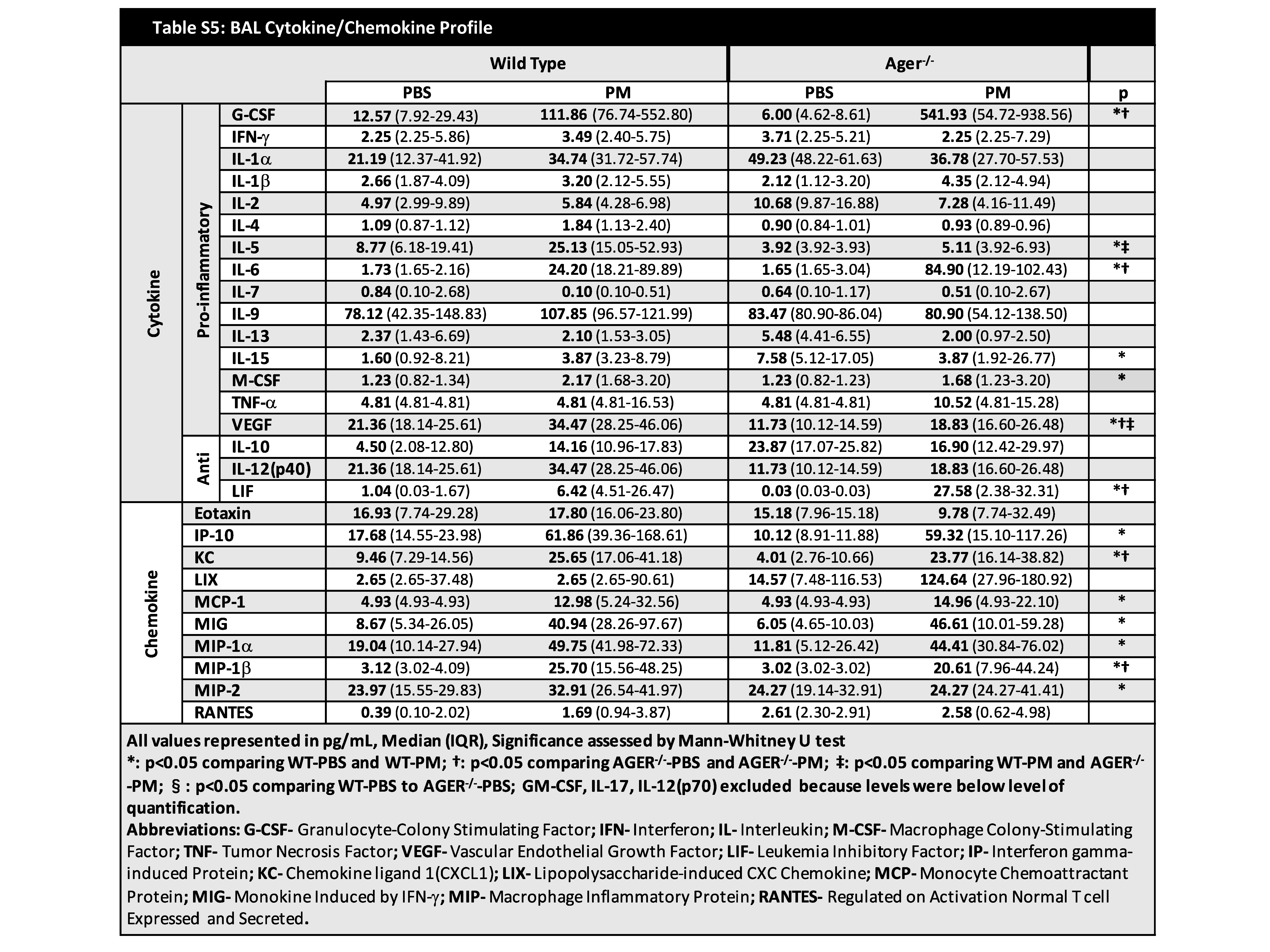

Supplement: S5 Table — (TIFF) [file pone.0184331.s005.tiff]

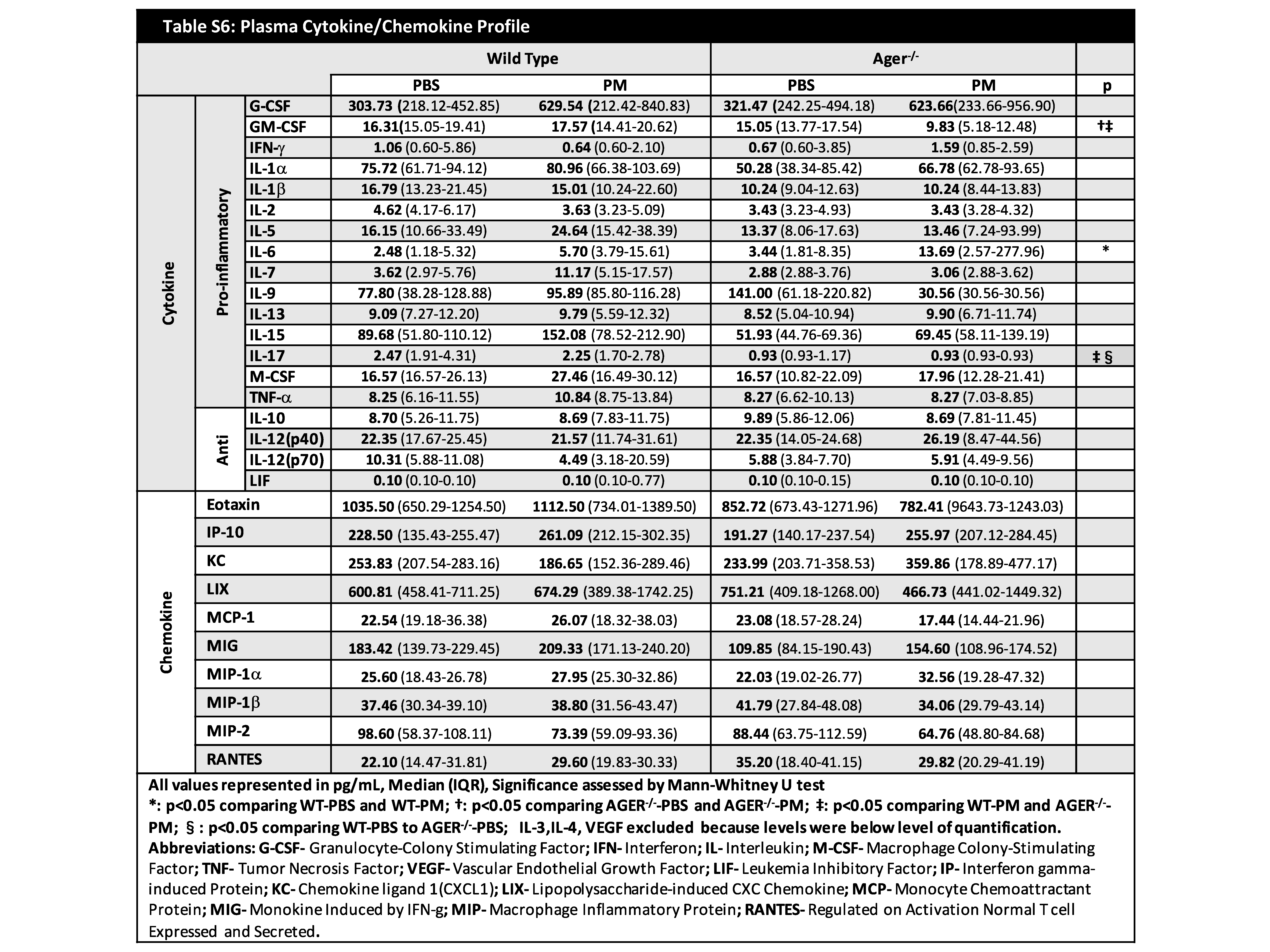

Supplement: S6 Table — (TIFF) [file pone.0184331.s006.tiff]

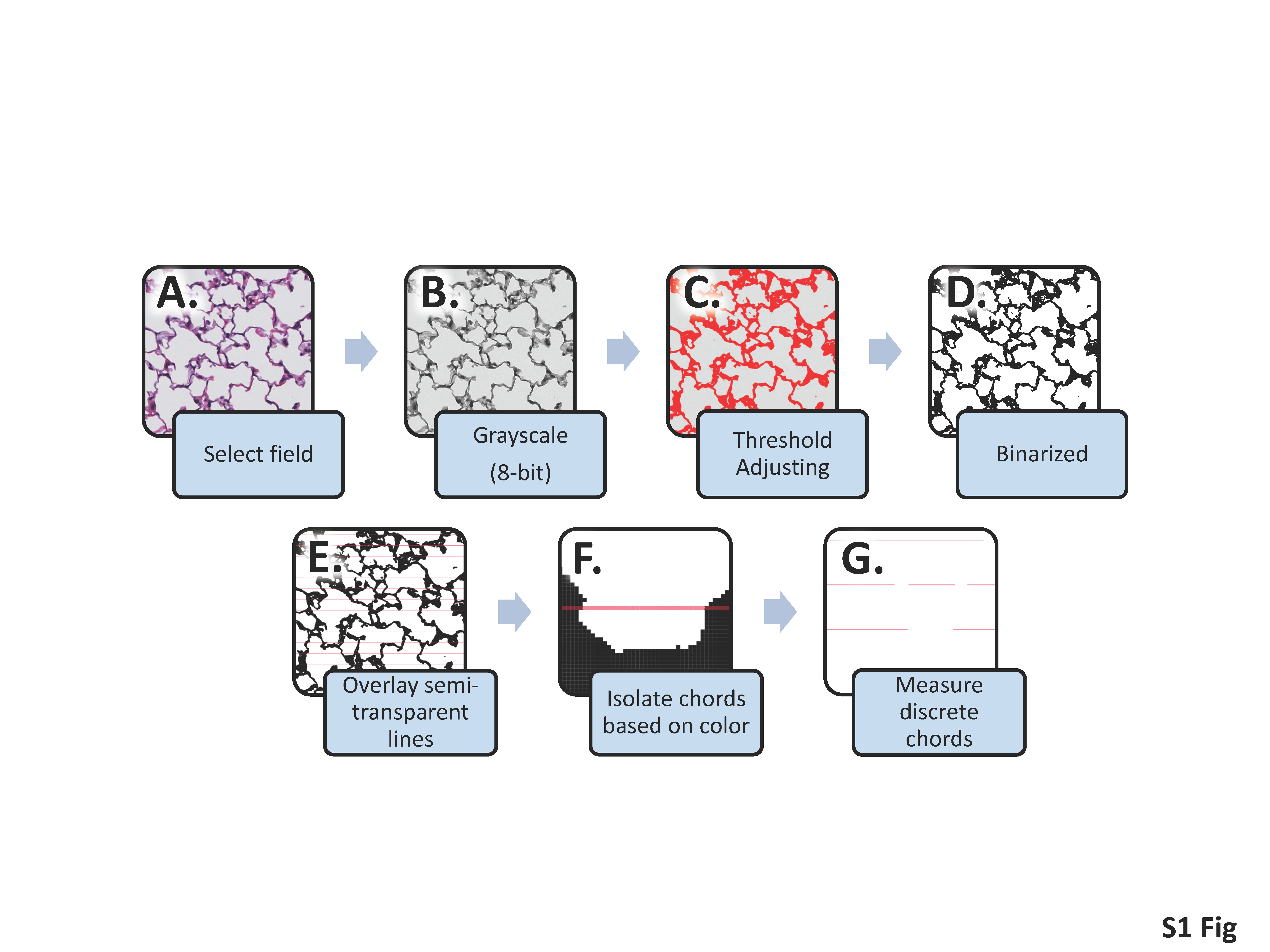

Supplement: S1 Fig — A. Select Fields. B. Converted to Grayscale (8-bit) C. Threshold Adjusted D. and binarized. E. Image was overlaid with 15 semi-transparent, horizontal test lines F. Discrete cords were isolated based on pixel color. This process was repeated for vertical test lines of the same spacing G. Chord lengths were measured. (TIFF) [file pone.0184331.s007.tiff]
